# Iron Oxide Nanoparticles Promote Cx43-Overexpression of Mesenchymal Stem Cells for Efficient Suicide Gene Therapy during Glioma Treatment

**DOI:** 10.7150/thno.60160

**Published:** 2021-07-13

**Authors:** Ai Li, Tianyuan Zhang, Ting Huang, Ruyi Lin, Jiafu Mu, Yuanqin Su, Hao Sun, Xinchi Jiang, Honghui Wu, Donghang Xu, Hongcui Cao, Xiaoyi Sun, Daishun Ling, Jianqing Gao

**Affiliations:** 1 Zhejiang Province Key Laboratory of Anti-Cancer Drug Research, College of Pharmaceutical Sciences, Zhejiang University, Hangzhou 310058, China.; 2 Department of Clinical Pharmacology, Key Laboratory of Clinical Cancer Pharmacology and Toxicology Research of Zhejiang Province, Affiliated Hangzhou First People's Hospital, Cancer Center, Zhejiang University School of Medicine, Hangzhou, 310006, China.; 3 Dr. Li Dak Sum & Yip Yio Chin Center for Stem Cell and Regenerative Medicine, Zhejiang University, Hangzhou 310058, China.; 4 Department of Pharmacy, the Second Affiliated Hospital, School of Medicine, Zhejiang University, Hangzhou 310009, China.; 5 State Key Laboratory for Diagnosis and Treatment of Infectious Diseases, the First Affiliated Hospital, School of Medicine, Zhejiang University, Hangzhou 310003, China.; 6 Department of Pharmacy, Zhejiang University City College, Hangzhou 310015, China.; 7 Cancer Center, Zhejiang University, Hangzhou, 310058, China.

**Keywords:** Mesenchymal stem cells, Glioma, Suicide gene, Gap junction, Iron oxide nanoparticles

## Abstract

**Background:** Mesenchymal stem cells (MSCs) have been applied as a promising vehicle for tumour-targeted delivery of suicide genes in the herpes simplex virus thymidine kinase (HSV-tk)/ganciclovir (GCV) suicide gene therapy against malignant gliomas. The efficiency of this strategy is largely dependent on the bystander effect, which relies on high suicide gene expression levels and efficient transportation of activated GCV towards glioma cells. However, up to now, the methods to enhance the bystander effect of this strategy in an efficient and safe way are still lacking and new approaches to improve this therapeutic strategy are required.

**Methods:** In this study, MSCs were gene transfected using magnetosome-like ferrimagnetic iron oxide nanochains (MFIONs) to highly express HSV-tk. Both the suicide and bystander effects of HSV-tk expressed MSCs (MSCs-tk) were quantitatively evaluated. Connexin 43 (Cx43) expression by MSCs and glioma cells was measured under different treatments. Intercellular communication between MSCs and C6 glioma cells was examined using a dye transfer assay. Glioma tropism and the bio-distribution of MSCs-tk were observed. Anti-tumour activity was investigated in the orthotopic glioma of rats after intravenous administration of MSCs-tk followed by intraperitoneal injection of GCV.

**Results:** Gene transfection using MFIONs achieved sufficient expression of HSV-tk and triggered Cx43 overexpression in MSCs. These Cx43 overexpressing MSCs promoted gap junction intercellular communication (GJIC) between MSCs and glioma cells, resulting in significantly inhibited growth of glioma through an improved bystander effect. Outstanding tumour targeting and significantly prolonged survival with decreased tumour size were observed after the treatment using MFION-transfected MSCs in glioma model rats.

**Conclusion:** Our results show that iron oxide nanoparticles have the potential to improve the suicide gene expression levels of transfected MSCs, while promoting the GJIC formation between MSCs and tumour cells, which enhances the sensitivity of glioma cells to HSV-tk/GCV suicide gene therapy.

## Introduction

Glioma is one of the most prevalent malignant tumours related to the central nervous system 1, 2. The prognosis of glioma remains a major challenge in the clinic due to its highly infiltrative, rapid proliferative and extensively invasive properties 3, 4. Surgical intervention is the main therapeutic strategy for glioma, yet its success is limited by the inability to eliminate all tumour foci apart from normal brain cells 5. Radiation therapy such as using γ-irradiation and chemotherapy like using temozolomide are the main adjuvant therapeutic strategies, but their outcomes in patients are limited, with a low median overall patient survival time accompanied by severe side effects 6-9.

Gene therapy is a novel strategy that has demonstrated promising potential in tumour treatment during the past decade. In particular, suicide gene therapy, such as herpes simplex virus thymidine kinase (HSV-TK)/ganciclovir (GCV) suicide gene therapy, is one of the most powerful strategies with a significant prognostic advantage in the treatment of malignant gliomas 10-12. The key to this therapeutic strategy is to enable specialised expression of HSV-tk at glioma sites to convert non-toxic GCV to its toxic metabolite, GCV-triphosphate (GCV-3P), and deliver GCV-3P for selected elimination of tumour cells through the bystander effect. Stem cells, such as mesenchymal stem cells (MSCs) have been developed as a vehicle for targeted delivery of suicide genes to tumours 13, 14. The excellent chemotaxis characteristics of MSCs toward the tumour microenvironment as well as other properties, including ease of isolation and expansion in culture 15, low immunogenicity 16, and the lack of ethical problems 17 make them ideal candidates to improve therapeutic outcomes and reduce the undesired toxic effects on normal cells. For example, we have demonstrated the superiority of using MSCs to carry HSV-tk for successful tumour-targeted suicide gene therapy 18, 19. Another typical example is a stereotactical administration of HSV-tk carried MSCs (MSCs-tk) in the contra-lateral brain of glioma can induce an efficient glioma inhibition 10. In addition, the treatment involving MSCs and HSV-tk did not injure normal brain cells due to the active glioma homing of MSCs, resulting in good safety 20, 21. Hence, MSCs have been regarded as a potential strategy to carry and deliver HSV-tk for suicide gene therapy to glioma with the assistance of GCV 22.

Despite the great success of HSV-tk/GCV suicide gene therapy using MSCs as carriers in the past several years, there are still many challenges for the practical application of this promising treatment strategy. The cytotoxicity of HSV-tk/GCV treatment to tumour cells is determined by the bystander effect 23, 24. Therefore, efficient intercellular delivery of the toxic GCV metabolite (GCV-triphosphate, GCV-3P) produced by HSV-tk enzymes in neighbouring cells is required 21, 25. Gap junction intercellular communication (GJIC) is believed to be the main approach to deliver the converted GCV metabolite to tumour cells 24, as it has a significant effect on the efficiency of the bystander effect to induce tumour cell apoptosis 26, 27. Connexin (Cx) 43 is the main Cx molecule comprising the GJIC channels and determines intercellular transfer efficiency through GJIC 10, 28. However, glioma cells exhibit relatively low Cx43 expression, thereby limiting the efficiency of the bystander effect 10, 29. A variety of methods have been developed to enhance GJIC function by up-regulating tumour cell Cx proteins, such as Cx43, for more powerful bystander cell death 10, 27-28, 30-31. However, transferring these experimental successes to practical treatment has been difficult. For example, a safe and efficient method to up-regulate the GJIC-involved connexions (Cxs) *in situ* is lacking and it is also difficult to ensure the high expression of the GJIC-involved Cxs in all tumour cells and avoid the risks of promoting GJIC of normal cells using current strategies. These challenges have limited the further application of this strategy for practical tumour treatment.

Recently, iron oxide nanoparticles (IONPs) have been demonstrated to possess the potential to trigger the overexpression of Cx43 after cellular uptake by cardiomyoblasts, which is attributed to the activation of JNK-c-Jun-mediated Cx43 expression pathways 32. Interestingly, the co-culture of MSCs with these Cx43-overexpressing cardiomyoblasts induces active cellular crosstalk. Thus, we herein propose a novel strategy of using IONPs to improve Cx43 expression of MSCs to augment the GJIC between MSCs and glioma cells rather than up-regulating Cx43 in glioma cells. These IONPs-harbouring MSCs may have active GJIC with glioma cells after homing to glioma tissues; thus, providing a facile strategy to augment the bystander effect compared with previous strategies of enhancing GJIC functions directly with tumour cells.

To prove this concept, previously reported magnetosome-like ferrimagnetic iron oxide nanochains (MFIONs) 33 were applied to genetically engineer MSCs for high expressions of HSV-tk suicide gene. Up-regulated expression of Cx43 was also detected in MSCs-tk, which promoted an improved GJIC with the co-cultured C6 glioma cells. Consequently, a significantly enhanced bystander effect on glioma cells induced by these MFIONs-harbouring MSCs-tk was observed, showing dramatically prolonged survival and decreased glioma sizes in glioma rats. To the best of our knowledge, the present study is the first report to show that magnetofection using IONPs enables the improved expression of suicide genes and assists with intercellular drug delivery through the GJIC promoted between MSCs and tumour cells *via* overexpressed Cx43 in MSCs. This novel strategy may provide a practical approach to augment the bystander effect of suicide genes carried by MSCs.

## Materials and Methods

### Reagents

MesenCult MSC Basal Medium and MesenCult Stimulatory Supplements were obtained from StemCell Technologies Inc. (Vancouver, Canada). Plasmid DNA coding luciferase enzyme (pGL-3) and Cx43-siRNA was obtained from Sangon Biotechnology Co. Ltd. (Shanghai, China). Plasmid DNA coding for HSV-tk was purchased from InvivoGen Biotechnology Co. (San Diego, USA). GCV was obtained from Haikou Qili Pharmaceutical Co., Ltd. (Hainan, China). 1,1'-Dioctadecyl-3,3,3',3'-tetramethylindocarbocyanine perchlorate (CM-Dil) was obtained from Invitrogen Life Technologies (Carlsbad, USA). Calcein acetoxymethyl ester (calcein-AM), 1% crystal violet and 4',6-diamidino-2-phenylindole (DAPI) were purchased from Yeasen Biotechnology Co. Ltd. (Shanghai, China). Dulbecco's modified Eagle's medium (DMEM) and foetal bovine serum (FBS) were purchased from Gibco BRL (Gaithersburg, USA). Iron (III) acetylacetonate (99%) and 4-biphenylcarboxylic acid (95%) were purchased from Acros Organics (Geel, Belgium). Polycaprolactone (MW = 14,000), oleic acid (90%), and branched polyethylenimine (PEI, MW = 25,000) were purchased from Sigma-Aldrich Chemical Co. (St. Louis, USA). Chloroform and tetrahydrofuran in chemically pure were purchased from Sinopharm Chemical Reagent Co., Ltd. (Shanghai, China).

### Cell culture

Human placental MSCs and green fluorescent protein (GFP)-expressing human placental MSCs (GFP-MSCs) were used and obtained according to our previous reports 33, 34. All handling was approved by the Research Ethics Committee of First Affiliated Hospital, School of Medicine, Zhejiang University (Reference number 2013-272). MSCs were cultured in a special medium containing MesenCult Human Basal Medium with 10% of MesenCult Stimulatory Supplements in a humid atmosphere of 37 °C and 5% CO_2_. C6 glioma cells were purchased from Shanghai Institute of Cell Biology and incubated with DMEM containing 10% FBS in a humid atmosphere of 37 °C and 5% CO_2_.

### Animals

Sprague-Dawley rats (male, weight 180-220 g) were purchased from Shanghai SLAC Laboratory Animal Co. Ltd. (Shanghai, China) and kept under specific-pathogen-free conditions. All animal experiments were approved by the Animal Experimental Ethics Committee of Zhejiang University and carried out in accordance with guidelines on animal handling.

### Preparation of MFIONs

The MFIONs were prepared following our previous report 33. Briefly, 25-nm ferrimagnetic iron oxide nanocubes were synthesised using the “heat-up” method and dispersed in an organic solution containing chloroform and tetrahydrofuran at a volume ratio of 1. Polycaprolactone (MW = 14,000) and oleic acid at a mass ratio of 2 were added to the mixture and stirred for 2 h. The same volume of the chloroform/THF mixture containing excess polyethyleneimine was added dropwise to the above reaction mixture with another 2 h of stirring. The modified nanoparticles were phase transferred to an aqueous solution through emulsification. Double-distilled water (ddH_2_O) was added to the chloroform/THF mixture containing the modified nanoparticles at a volume ratio of 5. The mixture formed a stable milky emulsion after sonication. Then the chloroform and THF were removed by rotary evaporation at 40 °C, and the free PEI was removed by washing with ddH_2_O. Finally, a certain volume of ddH_2_O was added to resolve the nanocubes for completing the phase transfer from oil-phase to water-phase, and these water-soluble nanocubes would self-assemble into 1-D nanochains (MFIONs) *via* the magnetic dipole interactions.

### Gene transfection using MFIONs-based gene complexes

Gene complexes based on MFIONs were freshly fabricated before application. Briefly, sterilised MFIONs were diluted in a 5% sucrose aqueous solution to an iron concentration of 15 μg/mL, which was incubated with 100 μg/mL pDNA in the same volume for 15 min at room temperature. Then, 130 μg/mL branched PEI at the same volume was added to the mixture for another 15 min to fabricate the gene complexes in a layer-by-layer formation. MSCs were seeded in a 24-well plate at a density of 6 × 10^4^ cells per well and cultured at 37 °C and 5% CO_2_ for 24 h. The culture medium was replaced with serum-free medium (500 μL) containing 40 μL of the gene complexes. The MSCs were incubated with MFIONs-based gene complexes for 4 h before replacement with fresh serum-containing medium. The MSCs were incubated for another 24 h before the evaluation experiments. Gene transfection efficiency was determined using a luciferase assay kit (Beyotime, Shanghai, China). Each experiment was repeated four times.

### Evaluation of the suicide effect

MSCs-tk were seeded in a 96-well plate at a density of 1 × 10^4^ cells per well. A GCV solution of 100, 200, 400 or 800 μg/mL was added to the medium for 5 consecutive days. Suicide efficiency was evaluated by cell viability of MSCs-tk, which was quantitatively detected with the Cell Counting Kit-8 (CCK-8) (Boster, Wuhan, China) assays. Live/dead assay using calcein-AM/propidium iodide (PI) double staining was also applied to evaluate the suicide effect.

### Estimation of the bystander effect

The viability of C6 glioma cells after co-culture with MSC-tk was determined to evaluate the bystander effect induced by MSC-tk. First, C6 glioma cells were labelled with GFP through gene transduction and screening according to our previous report 18. These GFP-expressing C6 glioma cells (GFP-C6) were co-cultured with MSC-tk at several cell ratios of 1:1, 5:1 and 10:1 (density of MSCs-tk was 1 × 10^3^ cells per well) in a 96-well plate. Different concentrations of GCV solution (100, 200 or 400 μg/mL) were added to the co-culture system for 5 consecutive days. The surviving GFP-C6 cells were quantitatively analysed *via* flow cytometry (BD Fortessa, BD Biosciences, San Jose, USA). GFP-C6 cells without co-culturing were regarded as the control to calculate the efficiency of the bystander effect.

In addition, the bystander effect was estimated after separating the MSCs-tk from the C6 glioma cells (cell number ratio: 1:1) using a 24-well Transwell plate (6.5 mm in diameter with 0.4 μm pore membranes, Corning Costar, Cambridge, USA). GCV solution (200 μg/mL) was added to the upper insert that was seeded with MSCs-tk. After 5 consecutive days of GCV treatment, the mortality of C6 glioma cells in the lower chamber was determined by a CCK-8 assay.

### In vitro expression of Cx43 determined by Western blotting

The* in vitro* Cx43 expression levels of MSCs-tk were determined by Western blotting assays according to a standard operating procedure. Briefly, cell samples were lysed in RIPA buffer (Servicebio, Wuhan, China) with 1 mM phenylmethylsulfonyl fluoride (Servicebio, Wuhan, China) and 5 μg/mL protease inhibitor cocktail (Servicebio, Wuhan, China). After centrifuging the cell lysates at 18,000 × g and 4 °C for 10 min, the sediments were resolved on 10% sodium dodecyl sulphate-polyacrylamide gel electrophoresis. The proteins were transferred to a polyvinylidene fluoride membrane (Millipore, Bedford, USA), followed by blocking with 3% bovine serum albumin (BSA) in TBST (10 mM Tris-Cl, pH 8.0, 150 mM NaCl, and 0.05% Tween-20) for 1 h. The Cx43 monoclonal antibody (ab79010, Abcam, Cambridge, UK) was applied as the primary antibody (1:1000, ddH_2_O) as reported previously 32 and incubated with the samples overnight at 4 °C. Goat anti-mouse IgG (Thermo Scientific Pierce, Rockford, USA) was used as the horseradish peroxidase-conjugated secondary antibody (1:5000, ddH_2_O) for 1 h incubation at room temperature. The bands were detected using the SuperSignal^®^ West Dura Extended Duration Substrate (Pierce, Bonn, Germany) and electrochemiluminescence (ECL) according to the manufacturer's instructions. Band densities were quantified by ImageJ software (ij152-win-java8, National Institutes of Health, Bethesda, USA). To measure the Cx43 expression levels in C6 glioma cells after co-culture with MSCs-tk, 5 × 10^4^ GFP-C6 glioma cells were co-cultured with the same number of MSCs-tk in a 24-well plate. After the 24 h co-culture, the cells were collected by trypsinization and the GFP-positive C6 cells in the mixed cell samples were isolated using fluorescence activated cell sorting (FACS, MoFlo Astrios EQ, Beckman, Brea, USA) according to the green fluorescence signals, and the Cx43 expression levels were detected by Western blotting as described above.

### *In vitro* Cx43 expression observed by confocal laser scanning microscopy

The *in vitro* Cx43 expression level of MSCs-tk and C6 glioma cells was further monitored after incubation with an anti-Cx43 antibody (Boster, Wuhan, China) by confocal laser scanning microscope (CLSM; FV3000, Olympus, Tokyo, Japan), when the cell samples were seeded in the 15-mm glass-bottomed dishes (Nest, Wuxi, China). In brief, cell samples were fixed by using 4% paraformaldehyde followed with the incubation in 0.1% Triton for 30 min. Afterwards, goat serum are used to block the cell samples for 30 min and anti-Cx43 antibody (Boster, Wuhan, China) was used as the primary antibody for another incubation for 8 h. Then, FITC-labelled goat anti-mouse IgG (Thermo Scientific Pierce, Rockford, USA) containing 3% BSA was used as the secondary antibody for 1 h incubation at room temperature. At last, DAPI staining was performed following the manufacturer's instructions to visualize the cell nuclei. The fluorescence of FITC was detected by CLSM to observe the Cx43 expression levels.

C6 glioma cells were previously labelled by CM-Dil for the observation of Cx43 expression. These CM-Dil-labelled C6 glioma cells were then co-cultured with MSCs-tk engineered with MFIONs-based gene complexes (MSCs-tk (M@P)) at the cell number ratio of 1:1 in a 6-well plate (Nest, Wuxi, China). After 24 h, all cells were harvested and the CM-Dil-labelled C6 glioma cells in the mixed cell samples were isolated using FACS as described above according to the red fluorescence signals. These isolated C6 glioma cells were further cultured at 37 °C in 5% CO_2_ for 24 h. The following procedures of the immunohistochemical staining were the same as described above.

### Observation of the intercellular drug delivery through the GJIC

GJIC functions were evaluated between MSCs-tk and C6 glioma cells by observing the intercellular transfer of calcein-AM from MSCs-tk into C6 glioma cells. In brief, C6 glioma cells were stained with CM-Dil fluorescent dye following the manufacturer's instructions and seeded into a 24-well plate at a density of 5 × 10^4^ cells per well. Two hours after seeding the cells, the same number of MSCs-tk treated with 1 µM calcein-AM for 30 min at 37 °C were added to the C6 glioma cells and co-cultured for another 4 h. The intercellular transfer of calcein-AM was observed by CLSM and quantitatively analysed *via* flow cytometry.

### *In vitro* tumour tropism of MSCs-tk

Transwell plates with a diameter of 6.5 mm and 8-μm pore membranes (Corning Costar Corp., Cambridge, USA) were applied to evaluate *in vitro* glioma tropism of MSCs-tk. In brief, MSCs and MSCs-tk were seeded in the upper chambers at a density of 2 × 10^4^ cells per well. The lower chambers contained C6 glioma cells or 0.5% serum medium. The cells were cultured at 37 °C and 5% CO_2_ for 24 h, the MSCs passing through the membrane were fixed in 4% paraformaldehyde and stained with 1% crystal violet. The number of MSCs migrating to the lower membrane surface was counted under an optical field (×10) in four samples.

### *In vivo* bio-distribution of MSCs-tk after systemic administration

SD rats (weight 180-220 g) were initially established as glioma-bearing rats as described previously 35. Briefly, healthy rats were anaesthetised followed by intracranial injection of 1 × 10^6^ C6 glioma cells dispersed in 10 µL PBS into the left forebrain (3 mm lateral, 1 mm anterior to bregma, 5 mm depth from the skull surface). CM-Dil labelled MSCs and MSCs-tk were intravenously (*i.v.*) administered to normal, sham and glioma-bearing rats through the tail vein to observe the distribution using an *in vivo* imaging system (Maestro, Cri Inc., Woburn, USA). At indicated time points, the rats were sacrificed using halothane, and major organs, including the brain, heart, lung, liver, spleen, and kidney were collected for fluorescence imaging. Furthermore, GFP-MSCs transfected with HSV-tk using MFIONs-based gene complexes (GFP-MSCs-tk(M@P)) were applied to observe the distribution of MSCs vehicles in glioma cerebrum after systemic administration through the tail vein. Two days post the initial administration of GFP-MSCs-tk(M@P), the cerebra samples were harvested and sectioned to slices for observation using a fluorescence digital slide scanner (VS200, Olympus, Tokyo, Japan) after DAPI nuclear staining.

### Detection of the GCV concentration in glioma cerebrum after intraperitoneal injection

High-performance liquid chromatography-ultraviolet detection (HPLC-UV) was applied to analyse the concentration of GCV administered in the glioma cerebrum. In brief, GCV solution (100 mg/kg) was intraperitoneally (*i.p.*) administered to glioma rats. About 3 h post GCV administration, the rats were anesthetized and the cerebral samples were harvested for further homogenisation using a homogeniser (JXFSTPRP24, Shanghai Jingxin, Shanghai, China). Then, 1% sodium hydroxide solution was added to extract GCV from the homogenate, and the GCV concentration was determined by reversed-phase HPLC (Agilent series 1260, Agilent Technologies, Palo Alto, USA) with a Diamonsil® C18 5 μm column (150 × 4.6 mm, Dikma, Beijing China). The chromatographic conditions were the same as our previous report 18.

### Therapeutic evaluation of MFIONs-engineered MSCs-tk against glioma

Briefly, the glioma-bearing rats were randomly divided into six groups: the PBS group (only* i.p.* administration of PBS), the GCV group (only *i.p.* administration of 100 mg/kg GCV solution), the MSCs group (*i.v.* administration of 3×10^5^ MSCs and *i.p.* administration of 100 mg/kg GCV solution), the MSCs-tk (PEI) group (*i.v.* administration of 3×10^5^ MSCs-tk engineered by PEI (MSCs-tk (PEI)) and *i.p.* administration of 100 mg/kg GCV solution), the MSCs-tk (M+P) group (*i.v.* administration of 3×10^5^ MSCs-tk engineered by PEI with MFIONs incubation (MSCs-tk (M+P)) and *i.p.* administration of 100 mg/kg GCV solution) and the MSCs-tk (M@P) group (*i.v.* administration of 3×10^5^ MSCs-tk engineered by MFIONs-based gene complexes (MSCs-tk (M@P)) and *i.p.* administration of 100 mg/kg GCV solution). Therapeutic MSCs were systemically injected into these rats 7 days after intracranial injection of 1 × 10^6^ C6 glioma cells. GCV solution was given through intraperitoneal injection 48 h after MSCs administration and for 7 consecutive days. The rats were then sacrificed one day after the termination of GCV treatments. Major organs including brain, heart, lung, liver, spleen, and kidney were carefully collected and fixed in 10% neutral buffered formalin for 48 h. The fixed tissues were embedded in paraffin and cut into 6 μm sections. Nissl staining (G1436, Solarbio, Beijing, China) and hematoxylin and eosin (H&E) staining (G1120, Solarbio, Beijing, China) were applied to observe the area of glioma according to the manufacturer's instruction. In addition, the apoptotic cells within the glioma samples were determined by using the terminal deoxynucleotidyl transferase (TdT)-mediated dUTP tip probe (TUNEL) assay. A TUNEL immunohistochemical staining kit (ZK8004, ZSGB-BIO, Beijing, China) was performed to stain the brain samples following the manufacturer's protocol. The number of apoptotic cells was counted in the field (three high-power fields of each section sample from three rats) using a microscope (Eclipse Ti-s, Nikon, Tokyo, Japan) to calculate the apoptosis index.

### Preliminary safety evaluation

Healthy rats were randomly divided into three groups: PBS group (only *i.p.* administration of PBS), GCV group (only *i.p.* administration GCV solution at the dose of 100 mg/kg), MSCs-tk (M@P) group (*i.v.* administration of 3 × 10^5^ MSCs-tk (M@P) with subsequent *i.p.* administration of GCV at the dose of 100 mg/kg). The body weights of all rats were monitored ever day during the treatment, and after 7 days consecutive treatments, the blood samples and major organs of all rats were collected for histological examination including routine blood tests and H&E staining.

### Statistical analysis

All statistical analyses were carried out with GraphPad Prism version 8.0 (GraphPad Software; www.graphpad.com). The results are performed in triplicate or four times and are described with mean + standard deviation (SD). One-way analysis of variance (ANOVA) and two-way-ANOVA were performed to detect differences between more than two groups. *p* value < 0.05 was considered significant, *p* value < 0.01 was considered very significant.

## Results

### Sufficient suicide effect of MSCs post non-viral gene transfection using MFIONs

**Figure 1:**
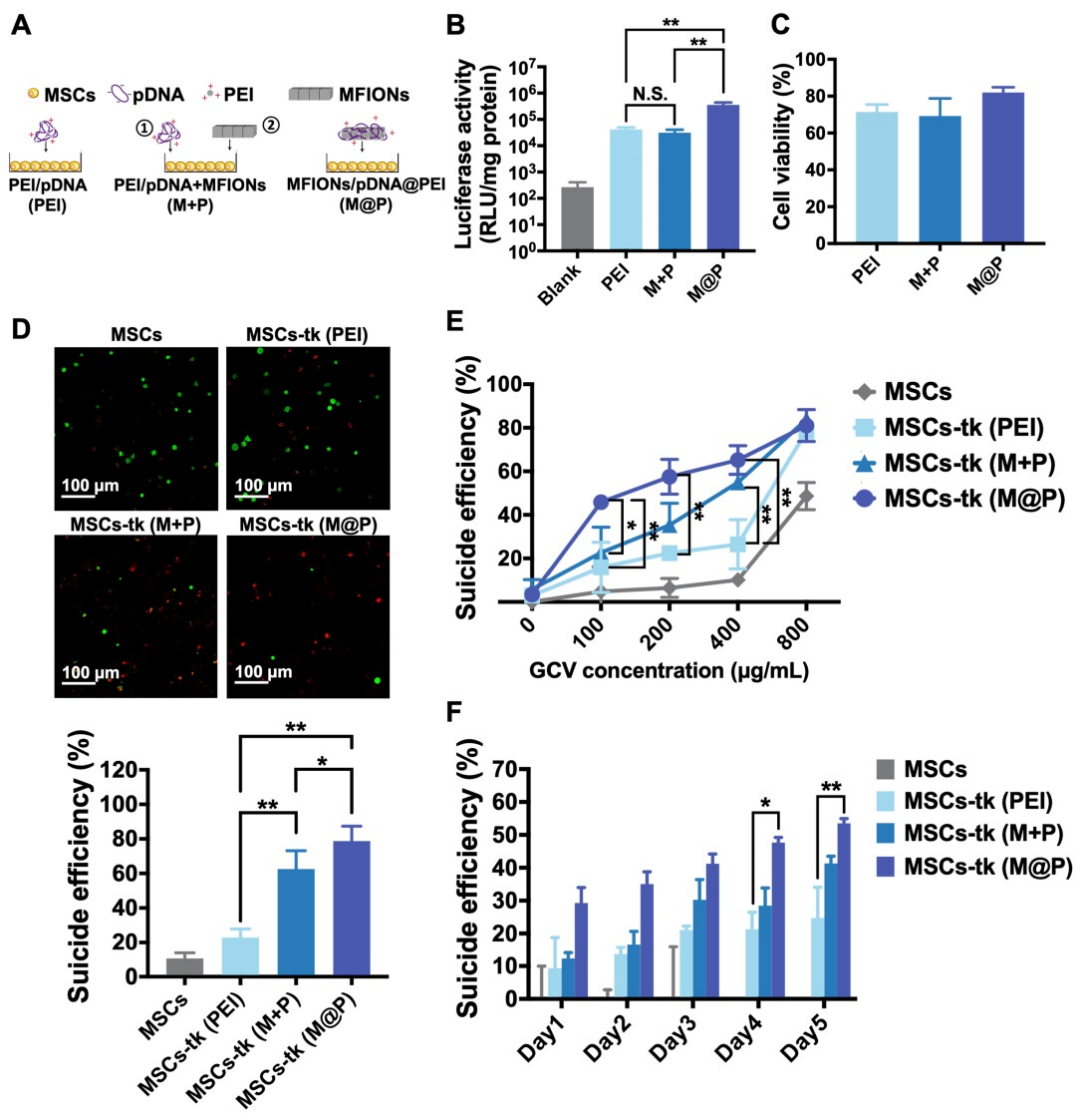
** Gene transfection of MSCs using MFIONs and the *in vitro* suicide effect evaluation.** A) Schematic illustration for gene transfection. Diverse preparations of the gene complexes including PEI/pDNA (PEI), PEI/pDNA mixed with MFIONs (M+P) and PEI/pDNA/MFIONs complexes (M@P) were applied for gene transfection of MSCs. Red “+” indicates a positive charge and green “-” indicates a negative charge. B) Luciferase activity of MSCs after gene transfection using pGL-3 reporter gene. C) Cytotoxicity evaluation of the gene transfection. D) Live/dead assays of suicide gene carried MSCs (MSCs-tk) treated with 200 μg/mL GCV for 5 consecutive days. The upper panel is the observations of the suicide cell death using confocal laser scanning microscope. Living cells are indicated by calcein-AM (green) and dead cells are indicated by propidium iodide (red). The low panel is the proportion of dead cells counted using ImageJ. E) Cell viability of MSCs-tk after treating with various concentrations of GCV for 5 consecutive days to determine the suicide efficiency. F) Suicide efficiency of MSCs-tk after treating with GCV at the concentration of 200 μg/mL on the indicated days. B, D, E) N.S. no significant difference, *p < 0.05, **p < 0.01, based on one-way ANOVA. F) *p < 0.05, **p < 0.01, based on two-way ANOVA. n = 4 for B) and n = 3 for C, D, E and F). Data are means ± SD.

Previously reported MFIONs-based gene complexes 33 fabricated with MFIONs, pDNA and free-PEI in a layer-by-layer manner (MFIONs/pDNA/PEI, M@P) were applied for the gene transfection of MSCs. The PEI-based gene complexes (PEI/pDNA, PEI) and MSCs initially transfected by PEI-based gene complexes followed with the incubation of naked MFIONs (PEI/pDNA+MFIONs, M+P) were added as a comparison (Figure 1A). To evaluate the gene transfection efficiency of M@P on MSCs, pGL-3 reporter genes were applied to fabricate gene complexes. As shown in Figure 1B, the highest luciferase activities were observed of MSCs transfected *via* M@P. The luciferase activities of MSCs transfected *via* PEI and M+P had no statistical difference, indicating the critical role of combining pDNA with MFIONs for the improved gene transfection efficiency. The results of cytotoxicity evaluations (Figure 1C) showed that the application of MFIONs had no detrimental effect on the viability of MSCs, indicating the relative safety of these IONPs to MSCs. The suicide effect of MSCs-tk with different engineering approaches was firstly visualized. MSCs and MSCs-tk were treated with GCV at the concentration of 200 μg/mL for 5 consecutive days and the viabilities were observed by the Live/dead assays as demonstrated in the upper panel of Figure 1D. The percentages of dead cells from the total cells were then calculated according to the live or dead signals, showing the most efficient suicide effect induced by MSC-tk (M@P) (the low panel in Figure 1D). Results of the cell viability further revealed that the suicide effect of MSCs-tk could be induced through a dose-dependent manner of the GCV concentration. An efficient suicide death of MSCs-tk was observed at the optimal GCV concentration of 200 μg/mL, while only slight toxicity to non-engineered MSCs (Figure 1E). Further increasing the GCV concentrations would induce non-ignorable cytotoxicity to MSCs. In addition, similar as the results of Live/dead assays, MSC-tk (M@P) showed the most efficient suicide effect. Moreover, the observation of the suicide effect at the indicated time points showed the constantly increased suicide cell death with the consecutive adding of GCV, and MSCs-tk (M@P) had the significantly highest suicide effect after the consecutive GCV treatment over 4 days (Figure 1F). Therefore, all these results verified the capability of MFIONs-based gene complexes to promote HSV-tk expression and augment the suicide effect.

### Significant augmented bystander cytotoxicity of glioma cells* in vitro*

**Figure 2:**
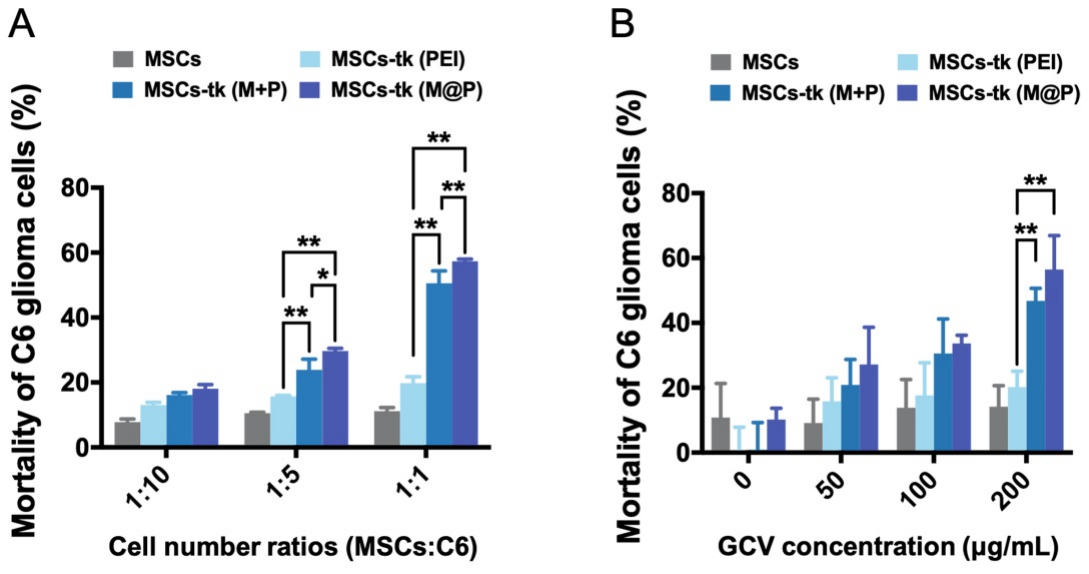
** Improved bystander effect against C6 glioma cells.** A) C6 glioma cells after co-culturing with MSCs were isolated using flow cytometry and their viability was determined. MSCs-tk transfected *via* MFIONs-based gene complexes (M@P) or PEI mixed with MFIONs (M+P) had a more efficient bystander effect than PEI transfected MSCs after treatment with 200 μg/mL GCV, particularly at a cell number ratio of 1:1. B) Both MSCs-tk transfected *via* MFIONs-based gene complexes (MSCs-tk (M@P)) and MSCs-tk transfected *via* PEI mixed with MFIONs (MSCs-tk (M+P)) achieved more effective tumour cell death through the bystander effect than PEI transfected MSCs (MSCs-tk (PEI)) at a cell number ratio of 1:1 post the GCV treatment at the concentration of 200 μg/mL. *p < 0.05, **p < 0.01, based on two-way ANOVA; n = 3 for A and B). Data are mean ± SD.

Except the effective activation of GCV to its toxic metabolite of GCV-3P by MSCs-tk, the intracellular delivery of GCV-3P from MSCs-tk to C6 glioma cells, which mainly relies on GJIC, is also critical for the bystander toxicity to inhibit the growth of C6 glioma cells. Here, GFP-C6 glioma cells were applied to evaluate the bystander effect of MSCs-tk by flow cytometry. As shown in Figure 2A, GFP-C6 glioma cells exhibited the lowest cell viability after being co-cultured with MSCs-tk (M@P) at cell number ratios of 1:5 and 1:1 in the presence of 200 μg/mL GCV. Notably, although PEI and M+P demonstrated similar gene transfection efficiency in Figure 1B, the bystander effect of MSCs-tk (M+P) was significantly higher than MSCs-tk (PEI), indicating the addition of MFIONs may also augment the bystander effect. Furthermore, the most efficient bystander toxicity to C6 glioma cells was observed when the GCV concentration was 200 μg/mL (Figure 2B). The efficiency of the bystander effect evaluated by total cell viability of the cell mixtures of C6 glioma cells and MSCs-tk showed similar results (Figure S1). All these results suggest that MFIONs improve the bystander effect of MSCs-tk on C6 glioma cells.

### Improved GJIC functioning between MSCs and glioma cells promoted by MFIONs

**Figure 3:**
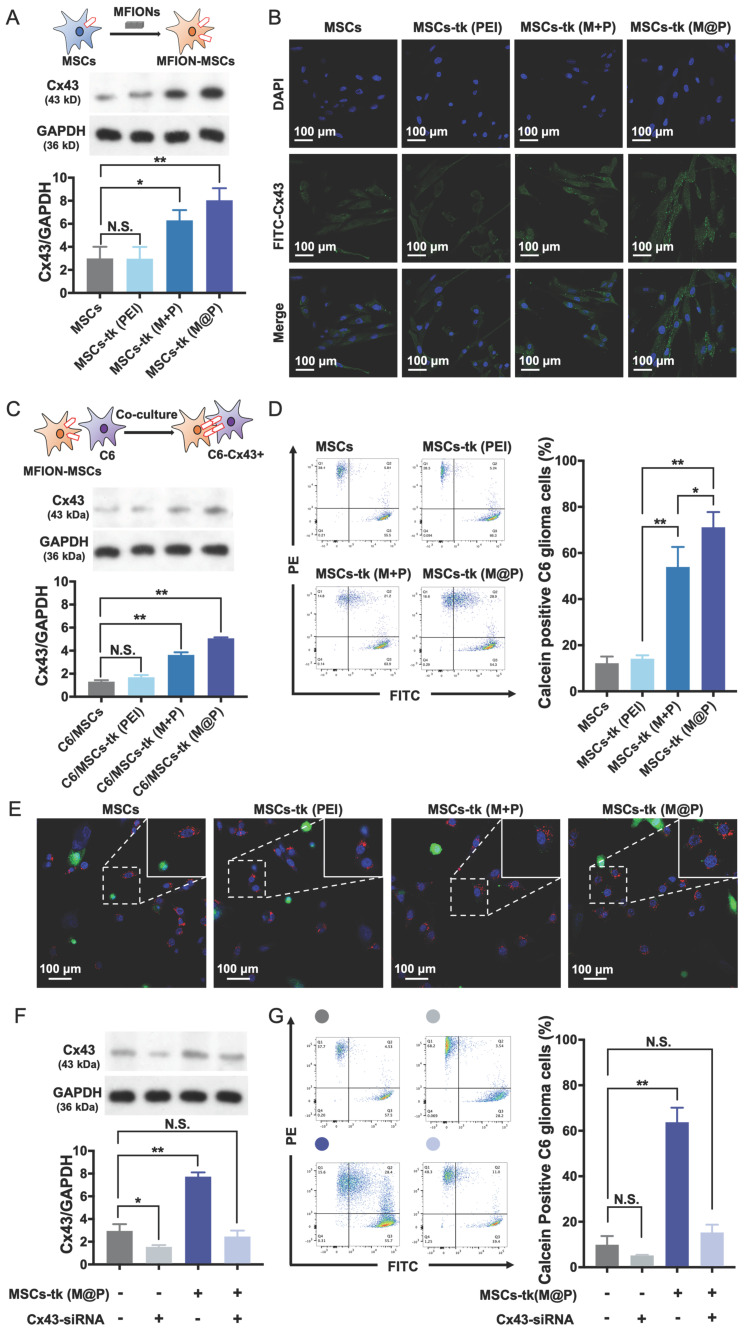
** Improved GJIC between MSCs and glioma cells triggered by MFIONs.** A) Cx43 expression levels of MSCs were determined by Western blotting assays. The semiquantitative data of the Western blotting were presented in the below. B) Images of Cx43 expression in MSCs taken by a confocal laser scanning microscopy. C) Cx43 expression levels of C6 glioma cells after co-culture with diverse MSCs determined by Western blotting assays. The semi-quantitative data were presented in the below. D) Efficiencies of intercellular communications between MSCs and C6 glioma cells *via* GJIC, indicated by dye transfer assays. E) Images of intercellular transfer of calcein from MSCs toward C6 glioma cells to visualize the intercellular communications. Blue: nuclei, green: calcein, red: CM-Dil labelled C6 glioma cells. The appearance of green signals in CM-Dil labelled cells (red) were regarded as the successful intercellular transfer (showed in the magnified images in the right top. F) Cx43 expression in MSCs post Cx43-siRNA transfection determined by Western blotting assays. G) Impacts of Cx43 on the intercellular communications functions. A, C, D, F, and G) N.S.: no significant difference, *p < 0.05, **p < 0.01, based one-way ANOVA. n = 3 for A, C, D, F, and G). Data are means ± SD.

HSV-tk gene therapy based on MSCs for glioma treatment relies heavily on an efficient bystander effect 10, 21. Overexpression of Cx43 plays a crucial role in GJIC and bystander effect 31, 36. Herein, Cx43 expression increased in MSCs engineered by M@P and M+P, while PEI had no such effect (Figure 3A). The highest Cx43 expression level was found in MSCs-tk (M@P). Furthermore, the Cx43 expressions in MSCs post indicated treatments were observed by CLSM after the immumohistochemical staining. Results revealed relatively a low expression level of endogenous Cx43 (green) in MSCs and MSCs-tk (PEI), while a higher Cx43 expression level was observed in MSCs-tk (M@P) and MSCs-tk (M+P) (Figure 3B). Next, the role of MFION-induced Cx43 overexpression of MSCs in the GJIC between MSCs and C6 glioma cells was studied. CM-Dil-labelled C6 glioma cells were co-cultured with MSCs or MSCs-tk for 24 h and then were isolated through FACS. Western blotting assays were performed to evaluate the Cx43 expression level in these C6 glioma cells. Interestingly, the expression level of Cx43 was also upregulated in C6 glioma cells 24 h after co-culture with MSCs-tk (M+P) or MSCs-tk (M@P) (Figure 3C). The Cx43 expressions in C6 glioma cells post co-culture with MSCs or MSCs-tk were also observed through the immunohistochemical staining (Figure S2). Furthermore, dye transfer assays were performed to evaluate the GJIC between MSCs and C6 glioma cells. MSCs and MSCs-tk were labelled with calcein-AM before co-culture. Calcein-AM became calcein in MSCs, which showed green fluorescence and could only be transferred through GJIC; C6 glioma cells were labelled with CM-Dil (red), which cannot pass through GJIC. Therefore, the efficiency of GJIC was calculated by determining the ratios of calcein and CM-Dil double labelled C6 glioma cells by flow cytometry. As demonstrated in Figure 3D, the highest ratio of calcein-positive C6 glioma cells was observed after co-culture with MSCs-tk (M@P). The ratio of calcein-positive C6 glioma cells also significantly improved after co-culture with MSCs-tk (M+P). These results suggest that the up-regulation of the Cx43 expression in MSCs can promote the GJIC efficiency between MSCs and C6 glioma cells. Observation of the intercellular delivery from calcein-AM endocytosed MSCs (green) toward CM-Dil labelled C6 glioma cells (red) *via* CLSM further confirmed these results (Figure 3E and Figure S3).

In addition, Cx43-silenced MSCs were prepared by inhibiting the Cx43 expression through Cx43-siRNA transfection to confirm whether the improved GJIC is activated by the Cx43 overexpression in MSCs. As shown in Figure 3F, the Cx43 expression levels of MSCs and MSCs-tk (M@P) were significantly inhibited after the transfection of Cx43-siRNA. Evaluation of the GJIC efficiency further showed that a significant augmented intercellular delivery of calcein toward C6 glioma cells was only caused by the Cx43-overexpressed MSCs (MSCs-tk (M@P)), indicating the overexpression of Cx43 in MSCs played a critical role to determine the efficiency of GJIC (Figure 3G). Moreover, it was further observed that the inhibition of Cx43 expression in MSCs-tk (M@P) would significantly decrease the bystander cytotoxicity (Figure S4). Separating the MSCs-tk (M@P) from C6 glioma cells using a Transwell plate with a diameter of 6.5 mm and 0.4-μm pore membranes sharply reduced the bystander effect on C6 glioma cells compared with the co-culture system (Figure S5). These results support the importance of Cx43 assembled GJIC for a highly efficient bystander effect, which relies on direct cell-to-cell contact.

Moreover, considering that the formation of GJIC relies on Cx43 expression in adjacent cells 37, the effects of Cx43 expression on C6 glioma cells were further studied. Reduced Cx43 expression levels in C6 glioma cells induced by siRNA transfection adversely affected intercellular drug delivery *via* GJIC (Figure S6) and reduced bystander cytotoxicity (Figure S7). These results in addition to the aforementioned results confirm the possibility of applying Cx43-overexpressed MSCs to promote GJIC function with C6 glioma cells for an augmented bystander effect.

### Highly efficient glioma homing of MFIONs-harbouring MSCs

**Figure 4:**
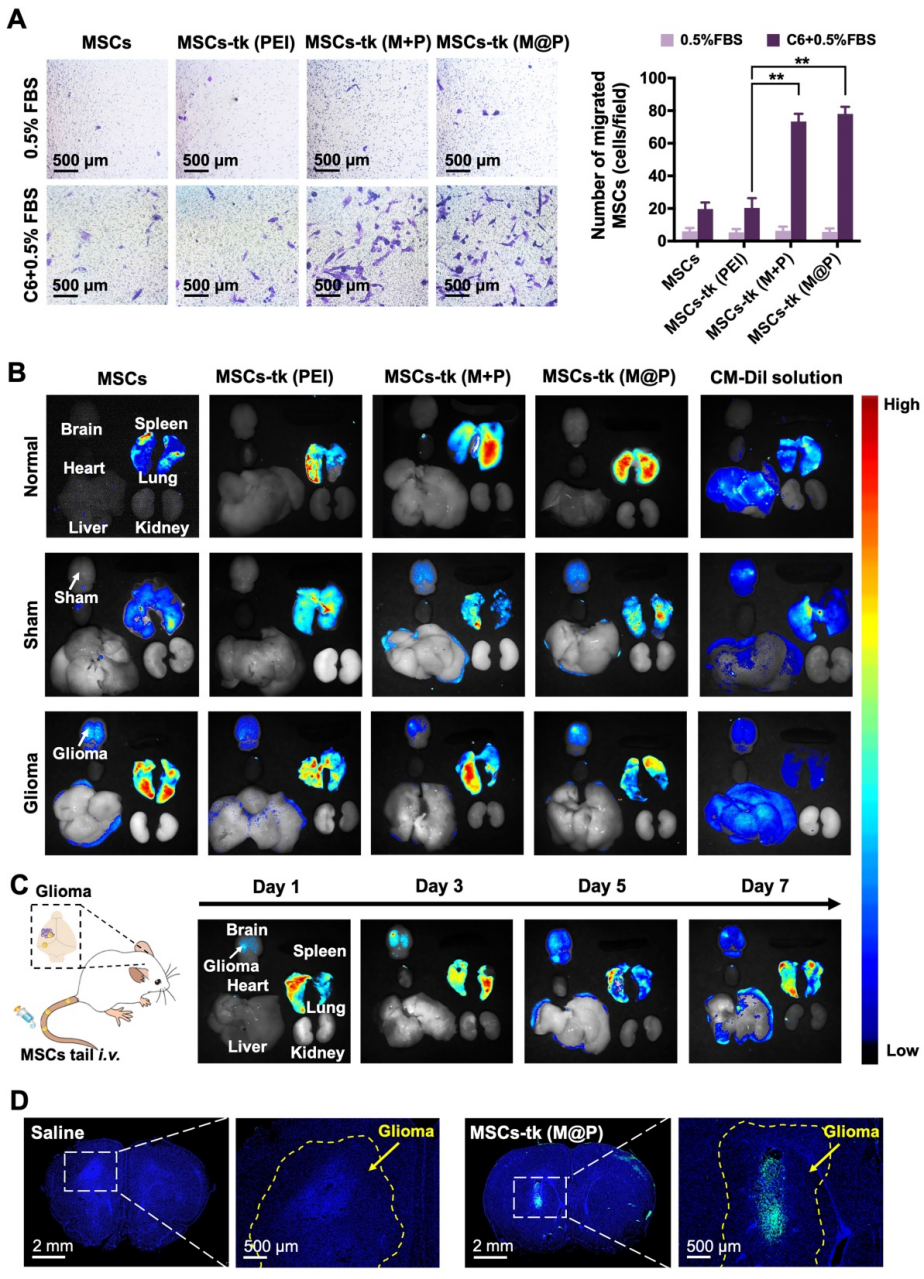
** Tumour tropism and distribution of MSCs post systemic administration through the tail vein.** A) Transwell migration tests to show the *in vitro* glioma tropism of MSCs. The migrated MSCs were stained in purple by crystal violet and their numbers were calculated from four random fields (showed in the left). Quantitative analysis of the migrated MSCs was showed in the right. B) Distribution of administrated MSCs (labelled by CM-Dil staining) in major organs of rats 48 h post *i.v.* administration. C) Long-term distribution of MSCs-tk (M@P) in glioma-bearing rats on the indicated days. MSCs-tk (M@P) were visualized by CM-Dil staining. D) Distribution of MSCs-tk (M@P) in glioma cerebra 48 h post *i.v.* administration. MSCs were labelled with green fluorescent protein (Green) and cell nuclei were stained by DAPI (blue). A) **p < 0.01, based one-way ANOVA. n = 4 for A). Data are means ± SD.

The homing of MSCs to tumour sites is crucial during glioma treatment to improve the concentrations of therapeutic agents and reduce side effects. The potential of MFIONs to promote stromal-derived factor-1α (SDF-1α) tropism of MSCs by up-regulating chemokine receptor type 4 (CXCR4) expression has been previously reported 33. Glioma sites secrete SDF-1α 38, suggesting that MFIONs-harbouring MSCs would show improved tropism toward gliomas.* In vitro* Transwell migration assays were performed to evaluate the tropism of MSCs-tk towards C6 glioma cells. As shown in Figure 4A, significant migration was detected when MSCs-tk (M@P) and MSCs-tk (M+P) were incubated with C6 glioma cells.

CM-Dil was applied to track the *in vivo* distribution of MSCs-tk post tail-vein injection. As shown in Figure 4B, 2 days after *i.v.* administration, MSCs and MSCs-tk mostly appeared in the lungs of normal rats, possibly due to MSCs lung entrapment 39, 40. MSCs and MSCs-tk (PEI) appeared in the lungs of the sham rats, while MSCs-tk (M@P) and MSCs-tk (M+P) appeared in the lungs and brains. In glioma-bearing rats, MSCs and MSCs-tk mostly appeared in the lungs and brains. Among them, MSCs-tk (M@P) and MSCs-tk (M+P) migrated to glioma regions. As shown in Figure 4C, MSCs-tk (M@P) first appeared in the lungs after *i.v.* administration, and gradually accumulated in the glioma regions on Day 3 after systemic administration. Notably, MSCs-tk (M@P) were observed to reside in the glioma regions until Day 7.

Furthermore, GFP-MSCs were applied to trace the distribution of MSCs-tk in the brains of glioma-bearing rats after *i.v.* administration. As shown in Figure 4D, high targeting efficiency to the glioma sites was observed 48 h after the *i.v.* administration of MSCs-tk (M@P), and these engineered stem cells even penetrated into the inside of the glioma. Interestingly, MSCs showed a weaker ability for the tumour penetration (Figure S8), which might attribute to the augmented homing capability caused by MFIONs according to our previous finding 33. To conclude, the MFIONs-harbouring MSCs possess the advantages for a highly efficient tumour targeting and tumour penetration.

### Therapeutic gains of applying MFIONs-harbouring MSCs for HSV-tk/GCV suicide gene therapy against glioma

**Figure 5:**
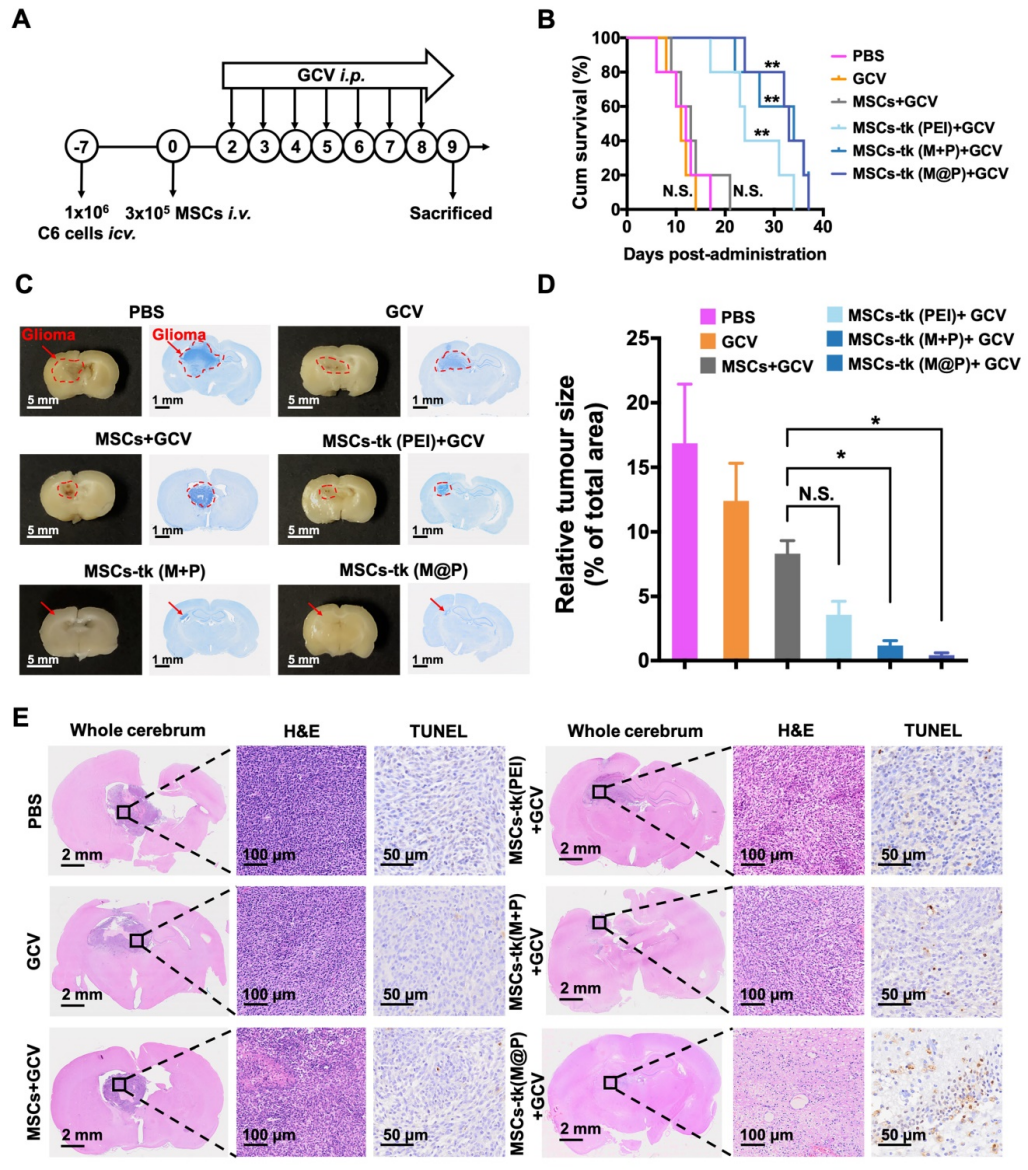
***In vivo* therapeutic gains of using MFIONs-harbouring MSCs for suicide gene therapy against glioma.** A) Schematic illustration to show the details of the therapeutic study. B) Survival curves of glioma rats after the different treatments. The differences between groups were compared with PBS group. C) Images of fixed brain samples and Nissl-stained brain sections. D) The areas of the gliomas were calculated with ImageJ according to the Nissl staining results. E) Pathological images of the cerebral sections (including H&E and TUNEL immunohistochemical staining). B) N.S.: no significant difference, **p < 0.01, compared to PBS group, based on Logrank. D) N.S.: no significant difference, **p < 0.01, based on one-way ANOVA. n = 5 for B). n = 3 for D). Data are means ± SD.

The *in vivo* anti-glioma effect of HSV-tk/GCV suicide gene therapy based on MFIONs-harbouring MSCs was assessed in glioma-bearing rats (Figure 5A). As shown in Figure S9, GCV was detectable in the brains of glioma-bearing rats 24 h after* i.p.* administration. GCV solution at 100 mg/kg was administered *i.p.* for 7 consecutive days 48 h post the injection of MSCs to reduce the side effects on healthy tissues according to the above distribution studies. As shown in Figure 5B, rats in the PBS group (12 days), the GCV group (11 days) and the MSCs+GCV group (13 days) had similar survival time. Prolonged survival time was observed when the glioma-bearing rats were treated with MSCs-tk (PEI), and the median survival time was 24 days. After being treated with MSCs-tk (M@P) or MSCs-tk (M+P), the median survival time increased to 33 and 34 days, respectively, demonstrating that Cx43-overexpressing MSCs-tk significantly ameliorated therapeutic efficiency. Additionally, the body weights of the PBS and GCV groups decreased gradually since Day 4 and the body weight of the MSCs+GCV group increased much more slowly than the other three groups from Day 4 to Day14 (Figure S10). After 7 days GCV treatment, the brain samples were harvested, fixed and Nissl stained to observe the sizes of the gliomas after the various treatments. As demonstrated in Figure 5C, a significant reduction in the glioma regions was observed after suicide gene therapy in the brain sections (left panel) and on Nissl staining (right panel). A semi-quantitative analysis was performed by measuring the area of the glioma region according to Nissl staining, which further verified the therapeutic outcome of MSCs-tk against C6 glioma (Figure 5D). In particular, the MSCs-tk engineered with M@P achieved the most powerful glioma inhibitory effect. These results were further confirmed by H&E and TUNEL staining demonstrated in Figure 5E. Notably, most cell apoptosis in the glioma region was observed after the MSCs-tk engineered with M@P treatment (Figure 5E). The number of the apoptotic cells was quantitatively calculated in Figure S11. All these results suggested that Cx43-overexpressed MSCs-tk showed enhanced therapeutic efficiency against glioma through efficient suicide effect and augmented bystander effect. The potential systemic toxicity of Cx43-overexpressed MSCs-tk was investigated through H&E staining of major organs. As shown in Figure S12, slight injury in lungs treated with MSCs-tk was observed in PEI group, M+P group and M@P group, suggesting that MSCs-tk entrapped in lungs may cause slight toxicity to glioma-bearing rats in this suicide gene therapy strategy.

### Preliminary histological safety evaluation of applying MFIONs-harbouring MSCs

**Figure 6:**
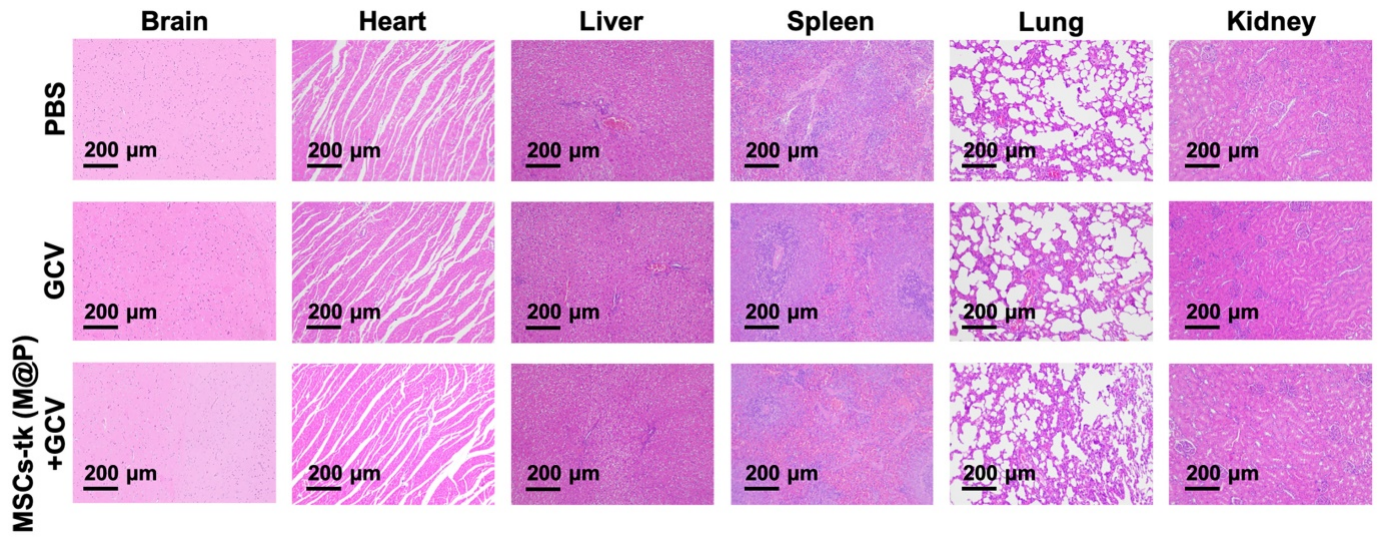
** Histological evaluation of the major organs after suicide gene therapy using MFIONs-harbouring MSCs-tk in healthy rats.** Representative H&E stained sections show the relative safety of MFIONs-harbouring MSCs-tk (MSCs-tk (M@P), regardless of a slight toxicity was observed in lungs.

The potential toxicity of HSV-tk/GCV suicide gene therapy based on MFIONs-harbouring MSCs was evaluated in healthy rats. The body weight (Figure S13) and routine blood tests (Table S1) suggested that there was no severe systemic toxicity of this therapeutic strategy. In addition, H&E staining of hearts, livers, spleens, lungs, kidneys, and brains showed relative safety of treating healthy rats with MSCs-tk (M@P) and GCV, as no remarkable abnormalities in these organs were observed (Figure 6). However, a slightly incrassate alveolar wall and a mildly increased lung interstitium were observed in the group after the treatment of MSCs-tk (M@P) and GCV, suggesting a slight pulmonary toxicity in healthy rats post this suicide gene therapy.

## Discussion

Suicide genes carried by MSCs for tumour-targeted delivery have been proposed as a promising strategy to improve the therapeutic efficiency and reduce the potential toxicity to healthy cells during suicide gene therapy for tumour treatment. Therefore, MSCs-applied suicide gene therapy has the potential to overcome the current challenges of glioma treatment 41. Briefly, the therapeutic efficiency of this strategy is affected by three major aspects: 1. The suicide gene expression level, which determines the efficiency of converting the nontoxic prodrug to the toxic metabolite; 2. The concentration of the prodrug delivered to the tumour tissues, which affects the amount of toxic metabolite at the tumour site; 3. The intercellular delivery efficiency of the toxic metabolite from MSCs to tumour cells. Much effort has been made to enhance suicide gene expression, including viral and non-viral approaches 19, 42-44. Moreover, several studies including ours have shown the advantages of enhancing the accumulation of the prodrug in tumour tissues 18, 45. Nonetheless, only a few studies have investigated the critical role of transferring the toxic metabolite from the MSCs carrier to tumour cells, which largely determines bystander effect performance. Several studies have shown the important roles of Cx43-containing GJIC 10, 28 or Cx43-hemichannels 46 of transfer cell death signals from apoptotic to healthy cells, indicating that Cx43 expression may be critical for spreading apoptosis toward surrounding cells and inducing an efficient bystander effect. For example, the bystander effect of suicide gene therapy using HSV-tk/GCV would be strongly enhanced by transplanting Cx43-overexpressing C6 glioma cells 10, which provides a new thinking of improving the efficiency of suicide gene therapy against glioma. However, the difficulty of selectively up-regulating Cx43 expression in glioma cells *in situ* makes this strategy more like a proof-of-concept than a therapeutic solution for practical treatment. Inducing Cx43 overexpression in administered MSCs might be a more facile strategy for two reasons: First, injecting Cx43 overexpressing MSCs is a more reasonable practical treatment compared with transplanting Cx43 overexpressing glioma cells. Second, the tumour homing ability of MSCs makes it possible to apply Cx43 overexpressing MSCs to promote transportation of the toxic metabolite toward nearby glioma cells. The current results support our proposal that Cx43 overexpressing MSCs improve intercellular drug delivery toward C6 glioma cells, thereby significantly increasing the bystander effect (Figures 2 and 3).

Interestingly, Cx43 expression of C6 glioma cells was also up-regulated after the co-culture with MSCs-tk (M+P) or MSCs-tk (M@P) (Figure 3C), suggesting the reasons for the improved GJIC between MSCs and C6 cells. However, comparing with the Cx43 expression levels in MSCs-tk (M+P) and MSCs-tk (M@P), these induced Cx43 overexpression in C6 glioma cells was still low. So far, we have little knowledge about the detailed mechanisms regarding this phenomenon. But these results provide a potential way to augment the bystander effect of HSV-tk/GCV suicide gene therapy delivered by MSCs.

In the present study, we have shown excellent glioma homing of MSCs after systemic delivery, in which most of the migrating MSCs accumulated at glioma sites other than healthy brain tissues (Figure 4D). This excellent tumour-targeting ability may also attribute to the application of MFIONs. In our previous study, we have demonstrated that MFIONs can dramatically improve the homing ability of MSCs toward injured cerebra, because they up-regulate the homing-related chemokine receptor CXCR4 in MSCs 33. Therefore, the efficient tumour-homing of MFIONs-harbouring MSCs, in addition to the tumour-cells selected eradication of HSV-tk suicide gene therapy ensure the low risks of these strategies to healthy brain cells and other organs except the lungs (Figures 5E and S12). The lung toxicity is probably induced by the MSCs entrapment in lung after systemic delivery as demonstrated in Figure 4C. Such lung entrapment of MSCs is believed partly owing to the relatively larger cell size of MSCs than the pulmonary capillaries and the cell surface adhesion molecules, such as α4 and α6 integrins 40. Considering the generation of the bystander effect requires both the HSV-tk expression and a sufficient GCV concentration, strategies like modifications of the cell surface composition to decrease the lung entrapment of MSCs or using some nanocarriers to concentrate the GCV in glioma brain and decrease the GCV distribution in lung may be useful.

We have previously shown good biocompatibility of MFIONs to MSCs, and no cytotoxicity of these IONPs to MSCs was observed at the present concentration (Figure 1C). However, the effect of triggered Cx43 overexpression on maintaining stemness, immune regulatory capacity and other biologic properties require further investigation.

**Scheme 1:**
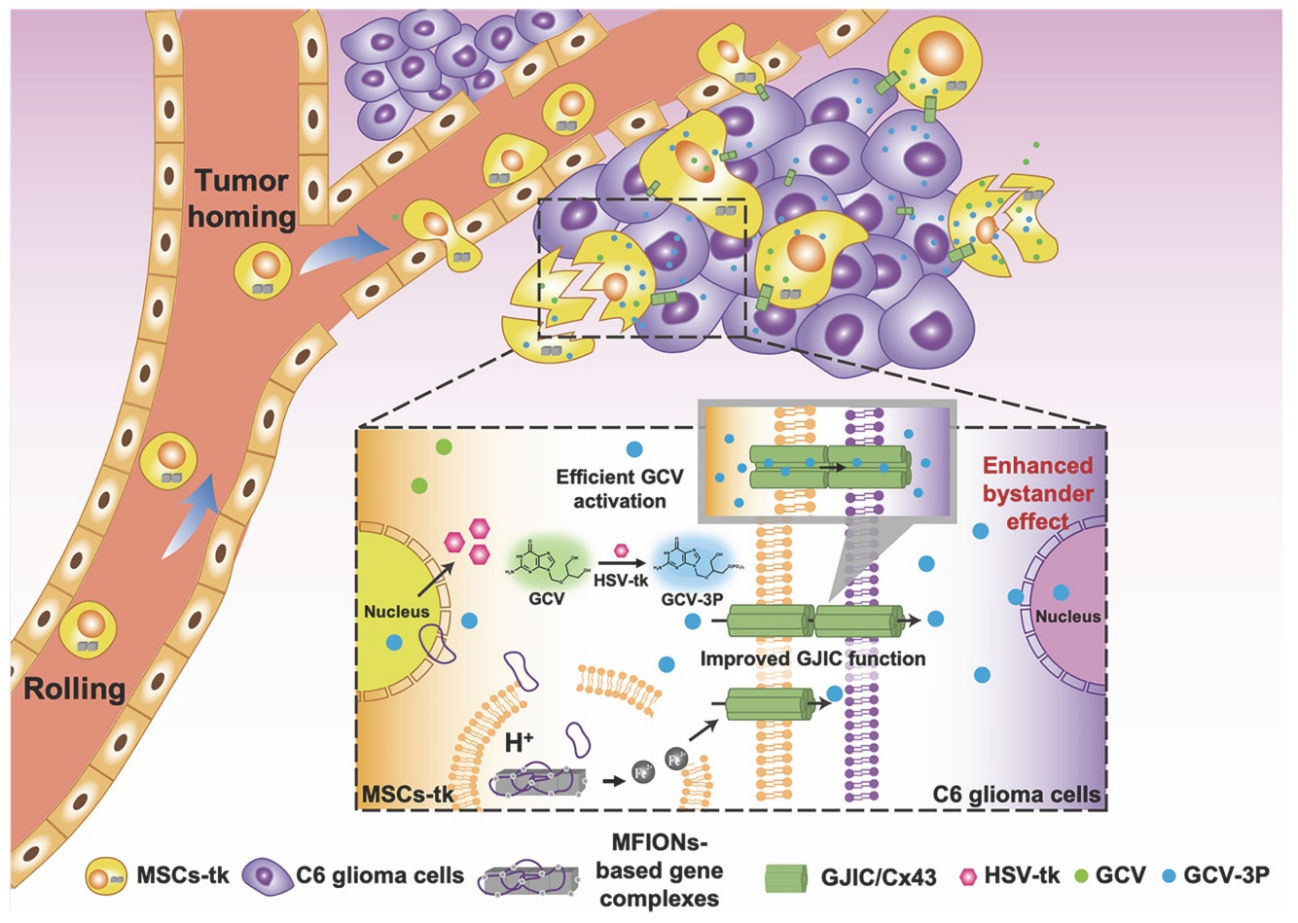
** Schematic illustration of using MFIONs to genetically engineer MSCs with HSV-tk suicide genes.** Excellent glioma homing and high HSV-tk expression enables an efficient convert of GCV to its toxic metabolite of GCV-triphosphate (GCV-3P) at the glioma sites. In addition, the overexpression of Cx43 triggered by MFIONs promotes the GJIC between MSCs and lioma cells, thereby enhancing the intercellular transfer of GCV-3P toward glioma cells and resulting in a significant bystander cell death of glioma cells.

To sum up, the present study shows a novel bio-application of IONPs to promote the GJIC of stem cells, which further enhanced intercellular communication between stem cells and tumour cells, benefiting the bystander therapeutic effect. Hence, IONPs are ideal candidates for stem cell engineering, as they improve the suicide effect with the efficient gene expression and augment the bystander effect through enhanced GJIC. Our study suggests that Cx43 overexpression in MSCs could also lead to a highly efficient bystander effect for glioma inhibition, providing a more facile approach for practical application of this robust suicide gene therapy (Scheme 1).

## Supplementary Material

Supplementary figures and table.
