# Supplementary material for: Iron Oxide Nanoparticles Promote Cx43-Overexpression of Mesenchymal Stem Cells for Efficient Suicide Gene Therapy during Glioma Treatment
Source: Theranostics. 2021 Jul 13;11(17):8254–69. doi: 10.7150/thno.60160 (PMC8344020; doi:10.7150/thno.60160)
Supplement: Supplementary file 1 — Supplementary figures and table. [file thnov11p8254s1.pdf]

Iron Oxide Nanoparticles Promote Cx43-Overexpression of Mesenchymal Stem Cells for Efficient Suicide Gene Therapy during Glioma Treatment

Ai Li[#], Tianyuan Zhang[#], Ting Huang , Ruyi Lin, Jiafu Mu, Yuanqin Su, Hao Sun, Xinchi Jiang, Honghui Wu, Donghang Xu, Hongcui Cao, Xiaoyi Sun, Daishun Ling, and Jianqing Gao[*]

[#]These authors contributed equally: Ai Li and Tianyuan Zhang.

*Corresponding author: e-mail: gaojianqing@zju.edu.cn

1. Supplementary Figures

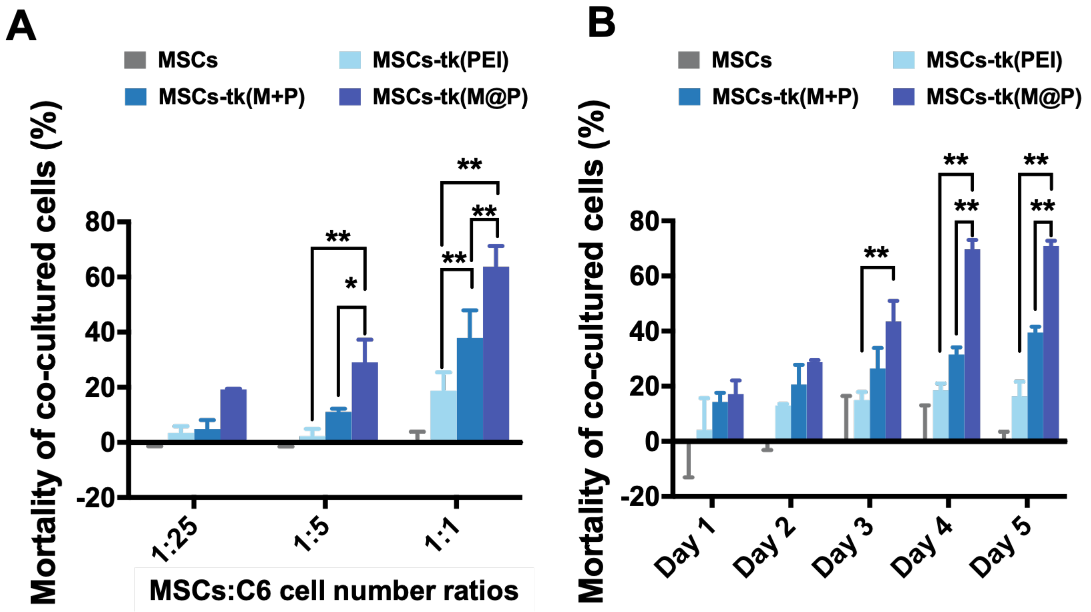

Figure S1 Bystander effect evaluated by the viability of co-cultured cells. A) Cell viability of co-cultured MSCs-tk and C6 glioma cells at different MSCs:C6 cell number ratios (1:25, 1:5, 1:1) after treating with GCV at the concentration of 200 µg/mL for 5 consecutive days. B) Cell viability of co-cultured MSCs-tk and C6 glioma cells at the cell number ratio of 1:1 after treating with 200 µg/mL GCV on the indicated days. A and B) $^*p < 0.05$, $^{**}p < 0.01$, based on two-way ANOVA. n = 3 for A) and B). Data are means ± SD.

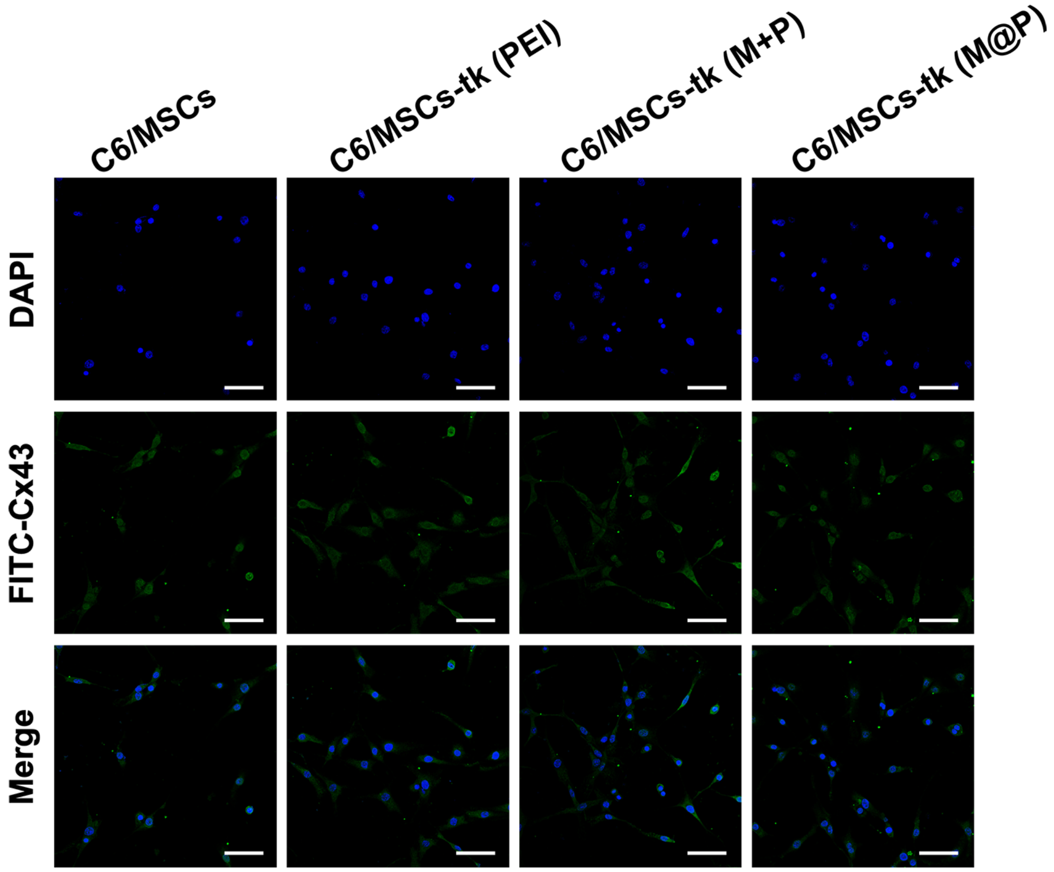

Figure S2 Cx43 expressions in C6 glioma cells after co-culture with MSCs for 24 h. The CM-Dil labelled C6 glioma cells were isolated after co-culture and the Cx43 proteins in C6 glioma cells were observed by a confocal laser scanning microscopy after immunohistochemical staining. Blue: nucleus, green: FITC-labelled Cx43 protein. Scale bars: 100 μm.

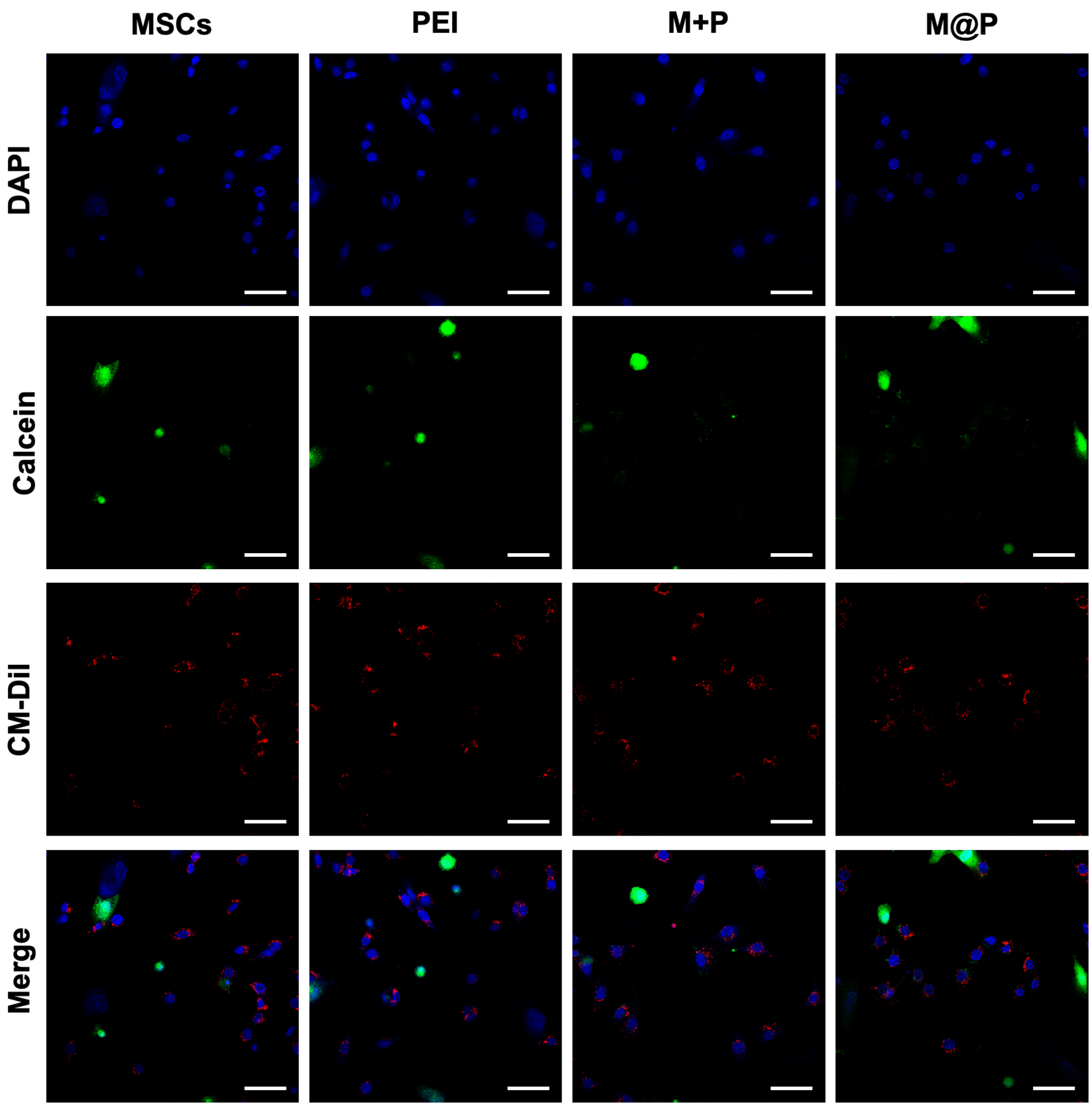

Figure S3 Images of the intercellular transfer of calcein from MSCs toward C6 glioma cells to visualize the intercellular communications. Images were taken by a confocal laser scanning microscopy. Blue: nucleus, green: calcein, red: CM-Dil labelled C6 glioma cells. Scale bars: 100 μm.

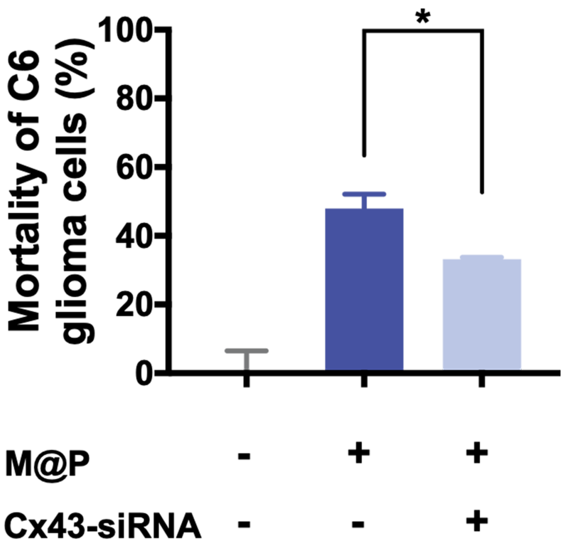

Figure S4 Cytotoxicity caused by the bystander effect on C6 glioma cells. MSCs with different treatments (no treatment, transfected with HSV-tk *via* MFIONs-based gene complexes (M@P) and both treated with M@P and Cx43-siRNA) were co-cultured with GFP-C6 cells at the cell number ratio of 1:1. The mortality of GFP-C6 cells were determined after 5 consecutive days treatments with GCV at the concentration of 200 μg/mL. *$p < 0.05$, based on one-way ANOVA. n = 3. Data are means ± SD.

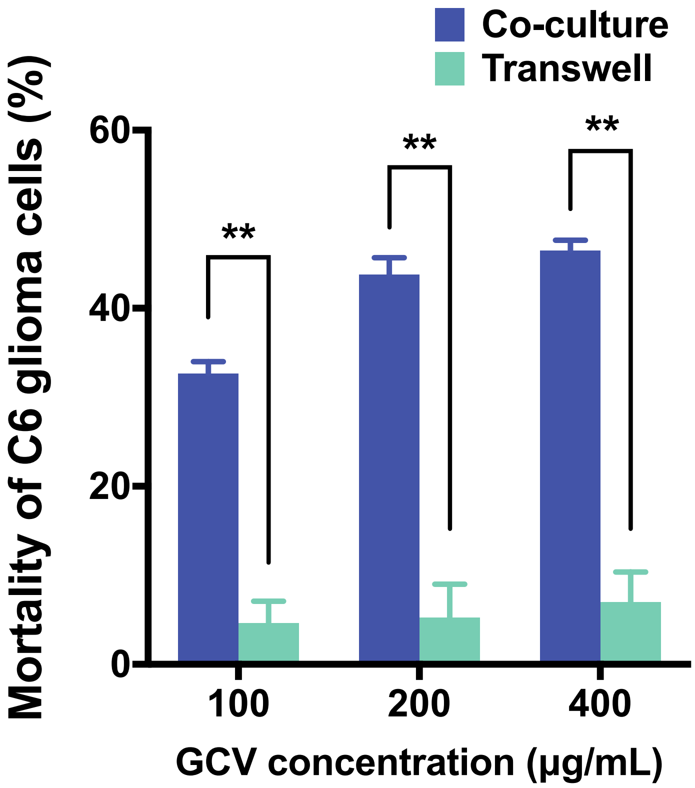

Figure S5 Efficiency of the bystander cell death in co-culture system and Transwell system. MSCs-tk transfected *via* MFIONs-based gene complexes (MSCs-tk(M@P)) were directly co-cultured with C6 glioma cells or separated cultured *via* Transwell plates at the cell number ratio of 1:1. The mortality of C6 glioma cells were determined after 5 consecutive days GCV treatments. **p < 0.01, based on two-way ANOVA, n = 3. Data are means ± SD.

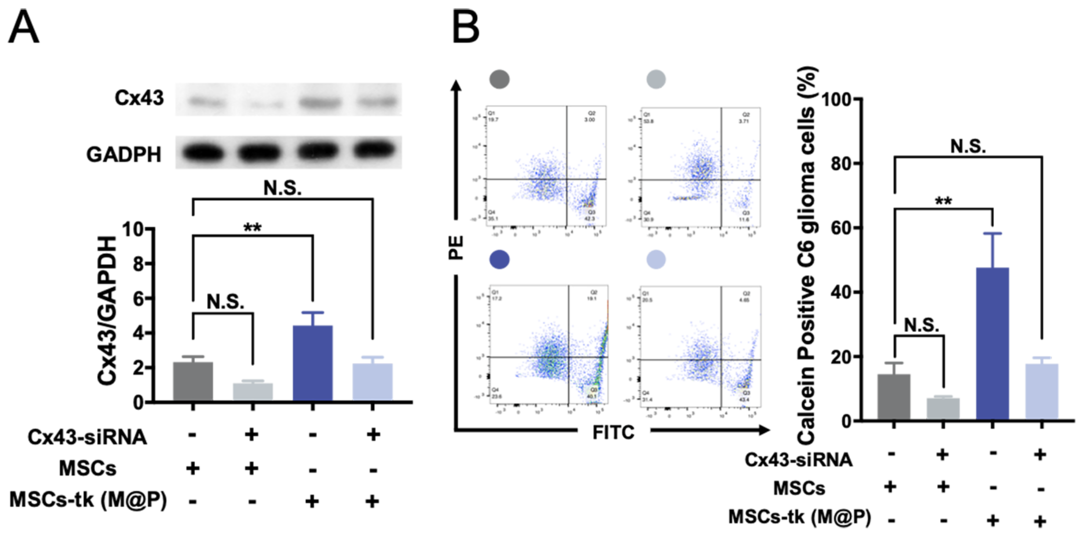

Figure S6 Imapcts of the Cx43 expression levels in C6 glioma clles on the gap junction intercellular communication. A) Western blotting assays were performed to evaluate the Cx43 expression levels of C6 glioma cells with different treatments. Cx43-siRNA indicates the Cx43 silenced siRNA, MSCs-tk(M@P) indicates the MSCs transfected using MFIONs-based gene complexes. B) Imapcts of the Cx43 expression levels of C6 glioma cells on the intercellular calcein transportation *via* GJIC. The ratio of the calcein-AM positive C6 glioma cells were determined by flow cytometer. N.S.: no significant difference, $^{**}p < 0.01$, based on one-way ANOVA. n = 3. Data are means ± SD.

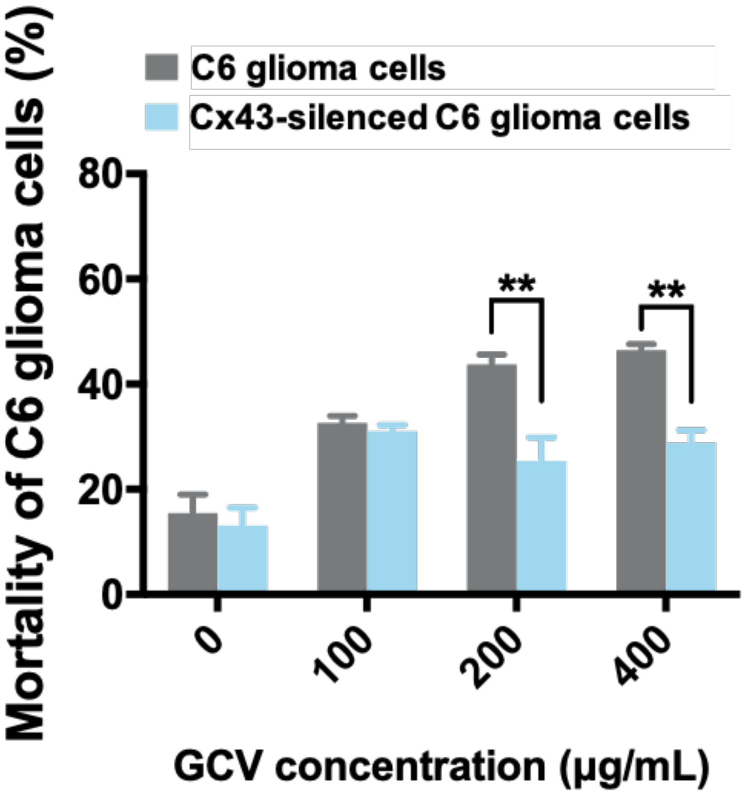

Figure S7 Impacts of the Cx43 expression by C6 glioma cell on the efficiency of bystander effect. MSCs-tk (M@P) were prepared by the gene transfection using MFIONs-based gene complexes and C6 glioma cells were treated with Cx43-siRNA to inhibit the Cx43 expression. **p < 0.01, based on two-way ANOVA. n = 3. Data are means ± SD.

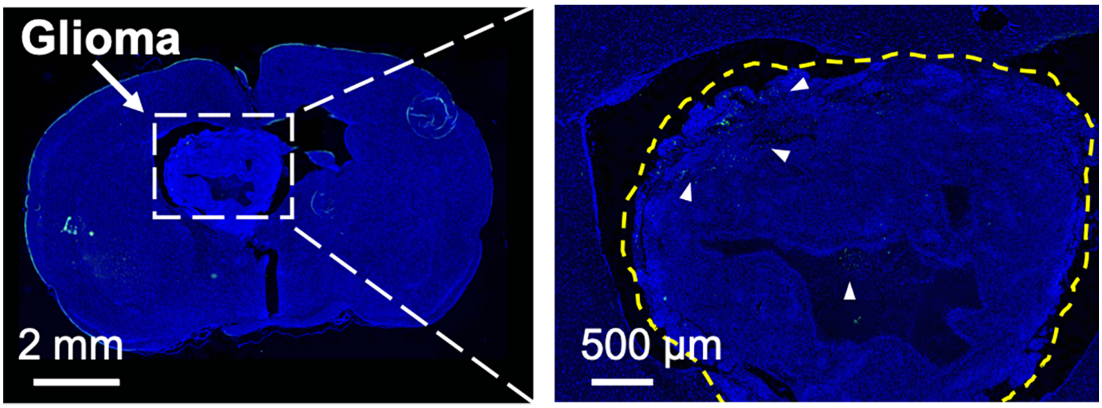

Figure S8 Fluorescent images of the distribution of GFP-lablled MSCs (GFP-MSCs) in glioma cerebrum 48 h after systemic administration. GFP-MSCs (white arrows) were observed to migriate to the area of glioma.

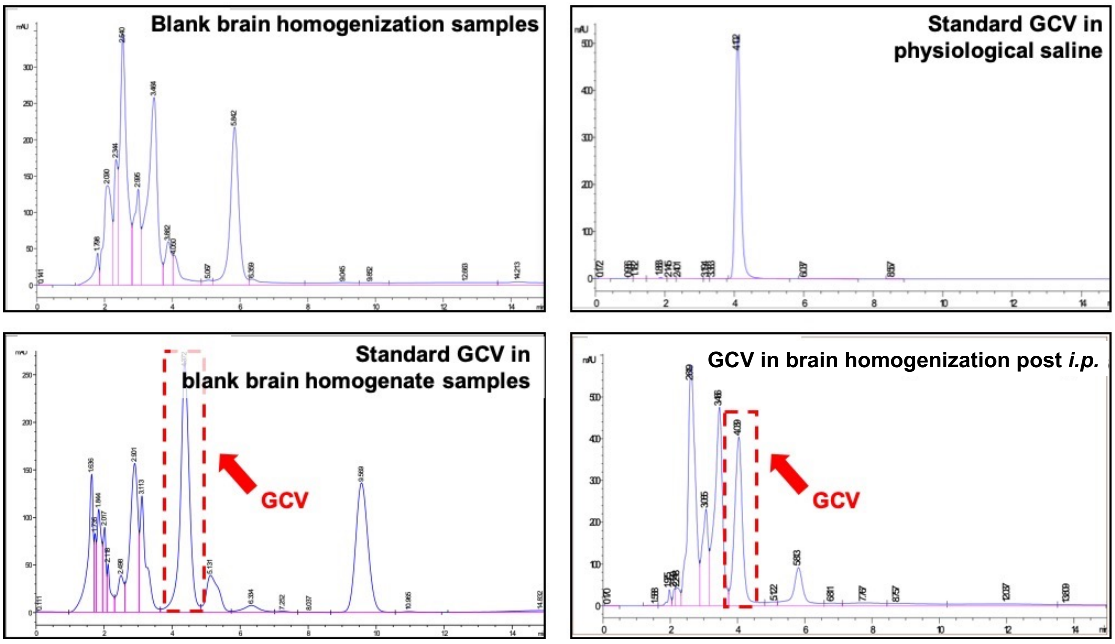

Figure S9 GCV concentration in glioma brain assessed by HPLC-UV method.

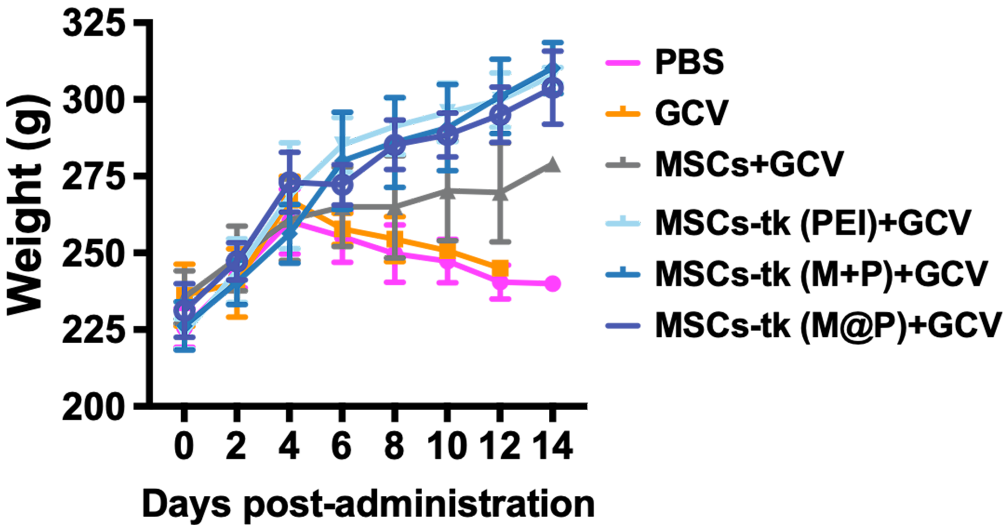

Figure S10 Body weights of glioma-bearing rats after different treatments. n = 5. Data are means ± SD.

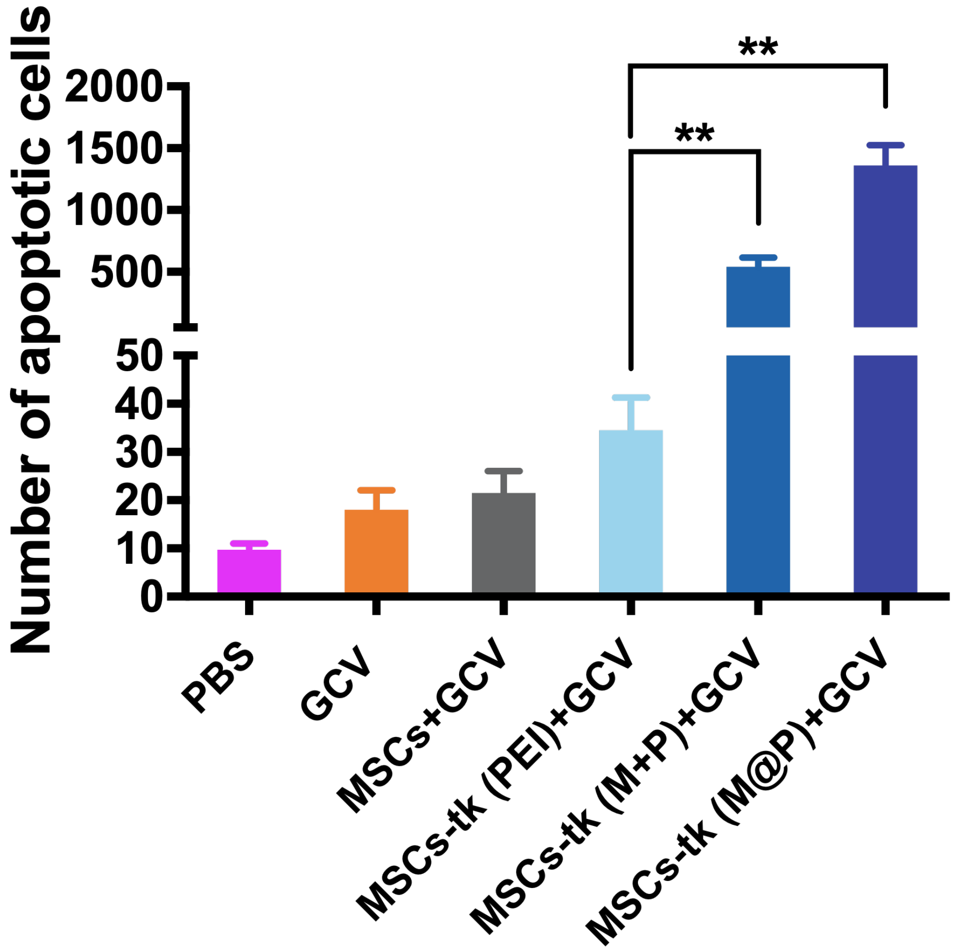

Figure S11 Quantitative analysis of the apoptotic cells according to the TUNEL staining. MSCs-tk (M@P) showed the most efficient bystander effect to induce the apoptosis of glioma cells. **p < 0.01, based on one-way ANOVA. n = 4. Data are means ± SD.

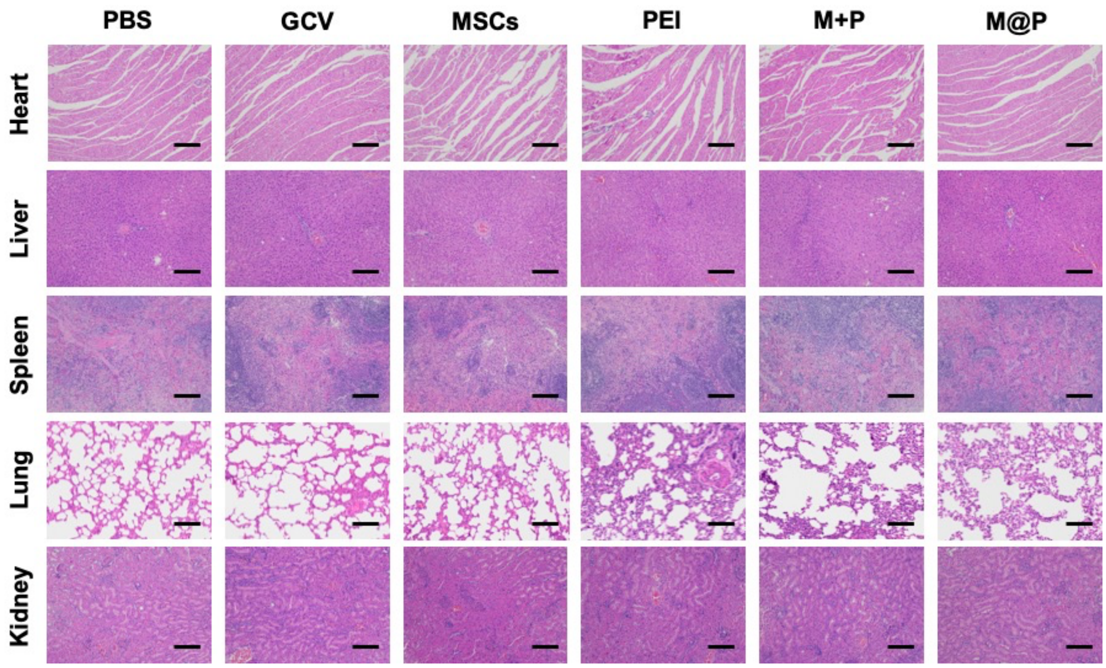

Figure S12 Haematoxylin and eosin stainging of major organs harvested from glioma-bearing rats after different treatments. No remarkable abnormalities in these organs were observed, except slight injury in lungs after the treatment of MSCs-tk (PEI), MSCs-tk (M+P) and MSCs-tk (M@P) with ganciclovir solution. Scale bars: 200 µm.

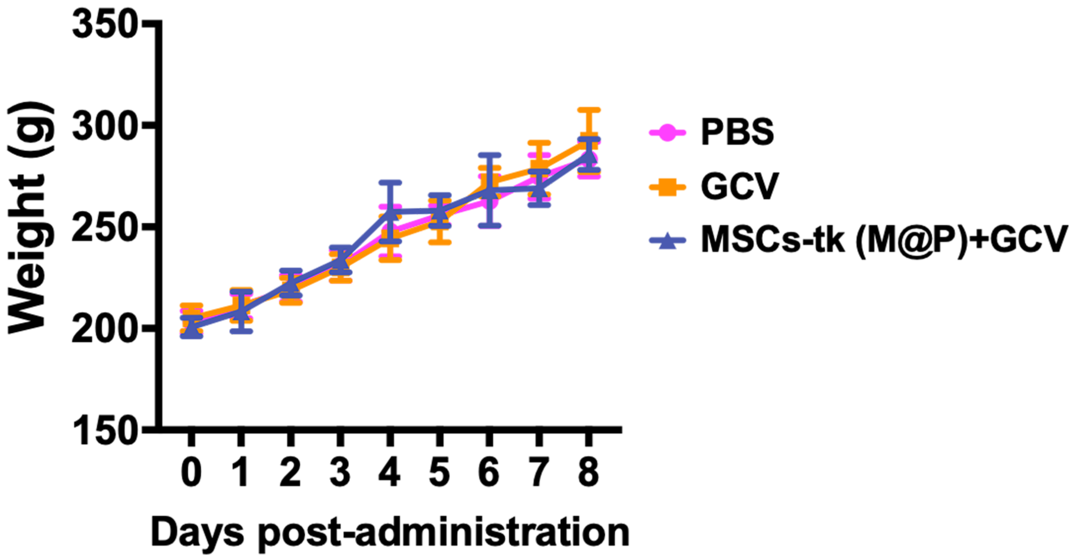

Figure S13 Body weight of normal rats in toxicity evaluation assays. n = 3. Data are means ± SD.

Table S1 Routine blood tests of normal rats with different treatments. *$p < 0.05$. based on one-way ANOVA. n = 3. Data are means ± SD.

Groups	Leukocyte		
	WBC (10^9/L)	NEU %	LYM %
PBS	10.67±0.28	21.53±7.55	56.93±13.00
GCV	13.22±1.57	23.47±1.68	58.47±6.89
MSCs-tk (M@P)+GCV	11.42±0.77	31.57±1.96	49.57±4.76
	**Red blood cell**		
	RBC (10^{12}/L)	HGB (g/L)	HCT %
PBS	6.16±0.12	130.00±1.00	33.90±0.20
GCV	6.55+0.15*	140.33±5.03*	34.53±0.31
MSCs-tk (M@P)+GCV	6.56±0.27	135.33±2.31*	31.97±1.95
	**Platelets**		
	PLT (10^9/L)	PCT %	MVP (fL)
PBS	491.33±222.04	0.47±0.22	9.50±0.61
GCV	477.67±113.07	0.47±0.12	9.90±2.64
MSCs-tk (M@P)+GCV	509.33±194.46	0.50±0.18	9.80±0.44

*Abbreviation: WBC (White blood cell), NEU (Neutrophil), LYM (Lymphocyte), RBC (Red blood cell), HGB (Hemoglobin), HCT (Hematocrit), PLT (Platelets), PCT (Platelet cubic measure distributing width), MVP (Mean platelet volume).
